# Impact of visualising healthcare quality performance: a systematic review

**DOI:** 10.1136/bmjopen-2023-083620

**Published:** 2024-11-02

**Authors:** Zijing Yang, Edward Alveyn, Mrinalini Dey, Nikita Arumalla, Mark D Russell, Sam Norton, James B Galloway

**Affiliations:** 1 Centre for Rheumatic Diseases, King's College London, London, UK; 2 Department of Psychology, Institute of Psychiatry, King's College London, London, UK

**Keywords:** Quality in health care, HEALTH SERVICES ADMINISTRATION & MANAGEMENT, Health Services, Quality Improvement

## Abstract

**Abstract:**

**Objective:**

Performance visualisation tools are increasingly being applied in healthcare to enhance decision-making and improve quality of care. However, there is a lack of comprehensive synthesis of their overall effectiveness and the contextual factors that influence their success in different clinical settings. This study aims to provide a broad synthesis of visualisation interventions not limited to a specific department.

**Design:**

Systematic review.

**Data sources:**

MEDLINE and Embase were searched until December 2022.

**Eligibility criteria:**

Randomised controlled trials (RCTs) and observational studies in English involving a visualisation intervention, either alone or as a core intervention, that reported quantitative outcomes including process and outcome indicators.

**Data extraction and synthesis:**

Data on study characteristics, intervention characteristics, outcome measures and results were extracted. The quality of evidence was assessed using the Grading of Recommendations Assessment, Development and Evaluation approach, and risk of bias was evaluated with Risk of Bias 2 for RCTs and Risk of Bias in Non-randomised Studies - of Interventions for non-randomised studies.

**Results:**

Of the 12 studies included, 2 were RCTs and 10 were observational studies, including 1 before-after study and 1 interrupted time series study. Five studies (42%) were conducted in teaching hospital settings. Compared with the control group or baseline, 10 studies reported a statistically significant change in at least one of their outcome measures. A majority of the studies reported a positive impact, including prescription adherence (6/10), screening tests (3/10) and monitoring (3/10). Visualisation tool factors like type, clinical setting, workflow integration and clinician engagement, may have some influence on the effectiveness of the intervention, but no reliable evidence was identified.

**Conclusion:**

Performance visualisation tools have the potential to improve clinical performance indicators. More studies with standardised outcome measures and integrating qualitative methods are needed to understand the contextual factors that influence the effectiveness of these interventions.

**STRENGTHS AND LIMITATIONS OF THIS STUDY**

- A broad scope of visual interventions was included instead of limited to one intervention, which allowed more comprehensive capture of the research in the field.
- Heterogeneity in included studies precluded the generation of an overall pooled estimate through meta-analysis.
- There is possible publication bias, as unsuccessful interventions might be less likely to have been submitted or accepted for publication.

## Introduction

 A growing number of quality improvement (QI) programmes have been implemented with the aim of improving healthcare quality, including the provision of appropriate therapies, avoidance of inappropriate tests and ensuring prompt diagnosis. Education,^1 2^ reminders,^3^ financial incentives,^4 5^ and audit and feedback are widely used as components of multifaceted QI interventions to modify clinical behaviour.^6^ Audit and feedback reports summarise the healthcare delivered by individual clinicians or teams over a set period.^6^ A Cochrane systematic literature review of 140 studies, published in 2012, found that audit and feedback can lead to small but potentially important improvements in clinician or practice professional performance and patient outcomes and may be more effective when given both verbally and in writing.^6^

In recent years, visual and digital tools have been increasingly used for audit and feedback which is part of QI programmes and offer a real-time view of performance, such as scorecards and dashboards, to monitor healthcare performance and delivery.^7 8^ Among these, dashboards have gained popularity as visual and interactive performance management tools, and have been employed in a wide range of clinical settings.^9^ For example, the use of dashboards in emergency departments has been shown to streamline workflow, with a visual representation of the department’s current status leading to improved coordination and reduced length of stay.^10 11^ However, the majority of such visualisation tools in clinical institutions are applied to monitor patients’ vital signs and record laboratory test results.^8^ So these applications failed to keep pace with the growing need for the improvement of healthcare providers’ performance.

The existing literature on visual and digital intervention primarily focuses on the development process and usability, such as more efficient comprehension of complex information and technical design of additional functions.^10 12 13^ Reviews which aim to investigate the effectiveness of visualisation interventions on improving care processes and outcomes have a focus only on individual intervention types (scorecard or dashboard)^14 15^ or settings (primary and aged care).^16 17^ There is a need for a comprehensive and timely review that evaluates the impact of various visualisation tools on clinician performance and care processes. Therefore, we conducted a systematic literature review to appraise the evidence for the effectiveness of interventions involving all visualisation tools of clinician performance display intended to improve process and outcome care (eg, prescription).

## Methods

### Data sources and searches

The following electronic databases were searched: OVID MEDLINE and Embase. Online supplemental appendix 1 contains the details of the search strategy. Search terms used included ‘dashboard’, ‘data displays’, ‘performance’, ’quality’, ‘clinician’ and their synonyms. We searched from these libraries respective inception dates and searches last updated on December 2022. The Preferred Reporting Items for Systematic reviews and Meta-Analyses (online supplemental appendix 2) and Synthesis Without Meta-analysis guidelines were followed in conducting this review^18 19^ (online supplemental appendix 3). Results were limited to the English language.

### Study selection

We included all studies that assessed data visualisation tools focused on healthcare professional performance, with the aim of improving healthcare quality. Visualisation interventions are frequently used as part of multifaceted interventions to improve and generate positive changes in healthcare outcomes, which can make evaluation of individual components challenging. As such, we only included studies that evaluated a visualisation intervention as a single or core intervention. Randomised controlled trials (RCTs) and observational studies were eligible for inclusion. Potential comparators considered were the preintervention baseline, no intervention, or any other intervention (single or multifaceted) not involving performance data display. Review articles, studies based on simulation and conference abstracts were excluded. The included studies were required to directly report, or provide sufficient information to derive, the following data elements necessary for standardisation: (1) Sample size, and (2) Quantitative outcome measurement including process and outcome indicators. Titles and abstracts of all identified studies were independently reviewed by three reviewers (ZY, EA and MD) using eligibility criteria. Studies included for full-text screening were also reviewed by independent reviewers. Disagreements were resolved through consensus discussion, with consultation of an additional reviewer if needed.

### Data extraction and quality assessment

Data were extracted on the author, country, year of publication, study aims, study design, intervention, implementation strategy, setting, population, sample size, duration, outcome measures and results. Data were extracted independently by two researchers (ZY and EA). Disagreement was resolved by consensus discussion. A Grading of Recommendations Assessment, Development and Evaluation approach^20^ was used to assess a summary of the overall quality of evidence. The included studies were assessed for their risk of bias using the Cochrane Risk of Bias 2 (RoB 2) tool^21^ for RCT studies and the Risk of Bias in Non-randomised Studies - of Interventions (ROBINS-I) tool^22^ for evaluating non-randomised studies.

### Data synthesis

We collected outcomes from all included studies and synthesised them in table 1. The outcome of our study was the difference in clinician performance between the control group or baseline measurement and the intervention group. All extracted outcomes of interest were binary but could be measured on different scales. When study outcomes were reverse scaled (ie, higher values indicated lower outcome performance rather than lower values), the effect size took the reciprocal. These dichotomous outcomes were expressed as relative risk (RR) using raw numbers collected from included papers. Due to the heterogeneity of the outcome measurements, no meta-analyses were conducted. A narrative approach was used to describe the evidence relating to the chosen outcome measures based on the direction and size of reported effects in accordance with Cochrane recommendations.^23^ Results were illustrated in a forest plot excluding an overall pooled effect estimate, using R software (V.4.2.2 R Project for Statistical Computing). An evaluation of heterogeneity using the I^2^ statistic was not possible due to the small number of included studies with a homogeneous study design and outcome. Heterogeneity was assessed instead through a visual and qualitative assessment.

**Table 1:** Description of included studies

Study (ref)	Study design	Country	Intervention	Study outcome	Participants	Include peer comparison or faculty performance or national benchmarks besides individual	Comparison /control
Lau *et al*^29^	Cohort	USA	Scorecard	Prescribing risk-appropriate VTE prophylaxis (increased from 89.4% at baseline to 95.4%)	Surgery residents	Yes	Usual care
Parks *et al*^32^	Cohort	USA	Dashboard	Adherence with clinical guidelines (increased from 66.5% to 84.6%)	Anaesthesiologist	Yes	Education
Banerjee *et al*^25^	Cohort	USA	Dashboard	Heart failure 30-day readmission (decreased from 14% to 10.1%)	Clinicians	Yes	Usual care
Linder *et al*^30^	RCT	USA	Dashboard	Antibiotic prescribing for ARIs (decreased from 47.22% to 46.54%)	Clinicians	Yes	Usual care
Inra *et al*^27^	Cohort	USA	Scorecard	Adenoma detection for male ≥25% (decreased from 31.9% to 30%)	Gastroenterologist	Yes	Usual care
Aboagye *et al*^24^	Cohort	USA	Dashboard	Surgical patients prescribed risk-appropriate VTE prophylaxis (increased from 95.6% to 98.4%%)	General surgical trainees	Yes	Usual care
Kadakia *et al*^28^	Before-after	USA	Scorecard	Utilisation of CT (decreased 33.33% to 25.01%)	Emergency department physician	Yes	Usual care
Yan *et al*^26^	RCT	USA	Dashboard	Antibiotic prescription (URI: decreased from 12.8% to 7.8%; bronchitis: decreased from 35.3% to 32.1%; sinusitis: increased from 76.7% to 76.8%; pharyngitis: decreased from 75.3% to 65.5%)	Clinicians	Yes	Education
Peek *et al*^33^	ITS	UK	Dashboard	Receipt of one or more potentially hazardous prescriptions (decreased from 2.61% to 1.65%)Inadequate prescription monitoring (decreased from 13.31% to 9.36%)	GP practices	Yes	Usual care
Twohig *et al*^34^	Cohort	USA	Dashboard	Colorectal cancer screening (increased from 54.42% to 59.83%)Patients with diabetes who had an A1c greater than 9% or no A1c in the past year (decreased from 35.08% to 33.62%)	GP practices	No	Usual care
O’Reilly-Shah *et al*^35^	Cohort	USA	Dashboard	Compliance with VT8 ventilation (increased from 59.3% to 65.3%)	Anaesthesiologist	Yes	Usual care
Meidani *et al*^31^	Cohort	Iran	Dashboard	Duplicated test (decreased from 13.6% to 7.19%)	Neurology residents	Yes	Usual care

A1c, glycated haemoglobin; ARI, acute respiratory infection; ITSinterrupted time seriesRCTrandomised controlled trialURI, urinary tract infection; VT8, tidal volume <8 mL/kgVTE, venous thromboembolism

### Patient and public involvement

None.

## Results

### Search results

**Figure 1:**
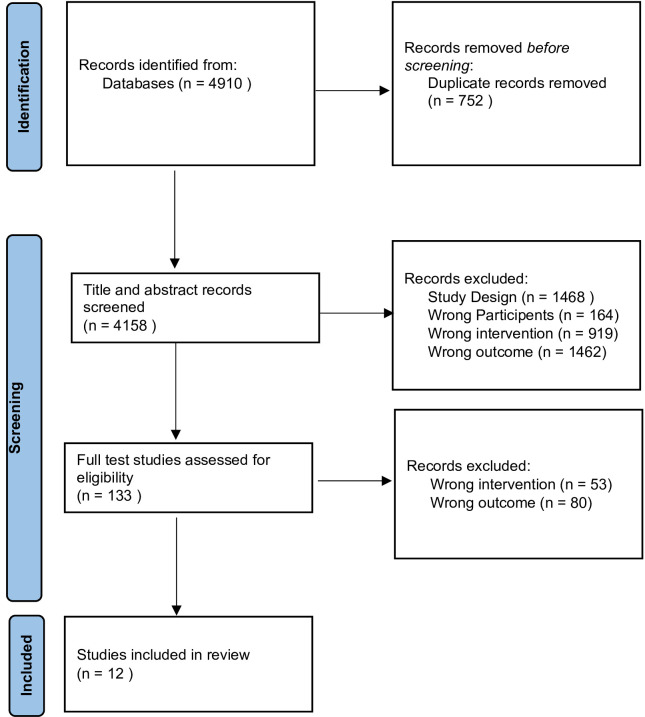
Flow chart of study selection,

A total of 4910 records were initially retrieved by searching electronic databases. Following deduplication, 4158 records remained. Twelve studies met the inclusion criteria and were included in this review.^24^,^35^ A flow diagram of studies is shown in figure 1.

### Characteristics of included studies

Of the 12 included studies published between 2000 and 2021,^24^,^35^ there are 2 RCT studies,^26 30^ and 10 observational studies,^2425 27^,^29 31^ including 1 before-after study^28^ and 1 interrupted time series study.^33^ The majority of eligible studies were based in the USA (n=10), one study^33^ was in the UK and one study was in Iran.^31^ Two studies were conducted at multiple hospital sites,^27 35^ four were in primary care^26 30 33 34^ and four were in tertiary care.^24^,^2631^ Five studies^24 25 28 29 31^ were conducted in teaching hospitals. Characteristics related to the population, interventions, comparison groups and outcomes for the 12 included studies are provided in table 1. More information about the study and intervention are supplied in online supplemental appendix 4.

### Risk of bias and study quality

We deemed two studies at moderate risk of bias using Cochrane’s RoB 2 tool for each RCT study. For the non-randomised studies, Cochrane’s ROBINS-I tool (online supplemental appendix 5) was used to conclude that five studies were at ‘low’ risk, one at ‘moderate’ risk and three at ‘severe’ risk of bias.

### Intervention characteristics

Three studies^27^,^29^ used a scorecard as a visualisation tool to provide the performance of healthcare providers, and nine studies^24^,^2630^ used a dashboard. The clinical departments in which the visualisation intervention was provided to clinicians included Anaesthetics, Gastroenterology, General Surgery, Primary Care, Emergency Medicine and Neurology. Ten studies^2425 27^,^31 33^ compared the intervention to usual care or no intervention, while two studies^26 32^ compared with an intervention of clinician education. More details about the intervention are supplied in online supplemental appendix 4.

### Outcome measures

The outcome measurements varied widely between studies, and some studies reported multiple primary outcomes. All included studies measured professional practice in process outcomes such as prescribing, use of laboratory tests or monitoring with dichotomous outcomes. Baseline or comparator performance was reported in all included studies. These reported outcomes were synthesised in table 1.

We used a pragmatic classification method to group studies with similar outcomes (online supplemental appendix 6). Based on the measures used in the included studies, we grouped study outcome measures relating to prescribing, use of laboratory tests or monitoring with dichotomous outcomes. Compared with the control group or baseline, 10 studies^24^,^2628 29 31^ reported a statistically significant change in at least one of their outcome measures, with prescription adherence the most reported success. Only one study indicated the effectiveness of the intervention dashboard among clinicians who actually used it,^30^ even though no significant differences were seen between dashboard users and non-users in the main analysis. The summarised effect of outcome on RR is shown in online supplemental appendix 6.

### Effectiveness of interventions on outcomes

In total, six studies^24 26 29 30 32 35^ reported on prescription behaviour change. There were two studies^26 30^ that examined changes in antibiotic prescribing. One study^30^ suggested that there was no difference between intervention and control practices in antibiotic prescribing for antibiotic-appropriate acute respiratory infection visits (65% vs 64%; p=0.68). However, among the users of the intervention, there was a significant difference in overall acute respiratory infection antibiotic prescribing rate (42% vs 50% for non-users; p=0.02) in subgroup analysis. Meanwhile, another study^26^ found a reduction in antibiotic prescribing for upper respiratory infections (12.8% vs 7.8%), bronchitis (35.3% vs 32.1%) and pharyngitis (75.3% vs 65.6%). Both Aboagye *et al*^24^ and Lau *et al*^29^ found that risk-appropriate venous thromboembolism prophylaxis prescribing practices improved following visualisation interventions (95.6% vs 98.4%, p<0.001; 89.4% vs 95.4%, p<0.001; respectively).

The comprehensiveness of investigation requests was assessed in four studies. Adenoma detection screening,^27^ CT utilisation,^28^ colorectal cancer screening^34^ and duplicated testing^31^ were used to assess clinicians’ adherence to diagnostic protocols. One study^27^ showed that recommended benchmarks for male adenoma detection rates (25%) were met postintervention but without improvement (31.9% vs 30.0%).

Additionally, rates of 30-day heart failure re-admissions,^25^ inadequate prescription monitoring,^33^ and patients with diabetes who had an HbA1c greater than 9% or no HbA1c in the past year^34^ were used to assess investigation adequacy as measured outcomes, where all showed significant improvements with effects favouring the intervention.

## Discussion

We conducted a systematic literature review on the effects of two distinct types of visualisation tools (dashboards and scorecards) on process and outcome indicators. Of the 12 publications that were included, 6 studies were published between 2020 and 2021, demonstrating the growing interest in developing tools that display and modify healthcare provider behaviour. The studies include a range of outcome measures, and the majority of these (10 of 12 studies) reported statistically significant improvements in clinician performance following visualisation interventions, with varying degrees of clinical benefit.

Visualisation interventions are frequently used as a part of multifaceted interventions to improve and generate positive changes in healthcare outcomes. This made evaluation of individual components challenging. Our review tried to overcome this barrier in isolating the effectiveness of the feedback display by only including studies that applied a display as a single or core intervention. Nevertheless, our results should be interpreted with caution as single interventions may be less successful when compared with multicomponent interventions.^36^ We might underestimate the true power of this intervention; that is, the observed effect might be due to the feedback display, the additional components of the intervention or a combination of them.

Previous systematic reviews in assessing the effectiveness of dashboard visualisation tools have not established clear links between this type of intervention and significant changes in clinician behaviour, due to the low number of high-quality and detailed research studies.^37^ In our own comprehensive search, we included all published studies of visualisation tools in healthcare settings, and summarised the effectiveness of process and outcome indicator intervention. This study demonstrates a growing literature supporting the likely effectiveness of these tools as half of the included studies in our review are published in the last 2 years. However, there remains a clear need for further higher-quality studies to provide an unambiguous case for dashboard visualisation tools.

In current practice, most hospital sites still use charts as a tool to display patients’ clinical information. Although an increasing number of sites use them as quality tools to measure performance indicators, these are diverse and not all of them quantitatively evaluate effectiveness. Thus we have tried to organise and facilitate the comparability of the efficacy of such interventions by categorising outcomes into three distinct strategies.

A recent systematic review of behavioural interventions in healthcare professionals found that interventions designed to compare individual behaviour to that of their peers were the most effective.^38^ However, in our review, all but one study presented peer performance comparisons in intervention data, and so we are unable to isolate this factor’s contribution in leading to greater improvements.

A previous study^39^ found that feedback display may be more effective when baseline performance is low, when the source of feedback is a supervisor or senior colleague, when feedback is provided more than once, when it is provided in both verbal and written formats, and when it includes both measurable targets and an action plan. However, in our review most included studies already had high compliance to standards at baseline. A recently published systematic review reported that in multicomponent interventions, dashboards facilitate more appropriate prescribing such as reduced opioid use for low back pain and antibiotic prescribing for upper respiratory tract infections.^37^ The studies evaluated in our review report a similar trend, where visualising the feedback of prescription performance may help improve treatment guideline adherence and prescription behaviours, such as more risk-appropriate prescribing, compared with prior reviews.

In some contexts, the use of dashboards appears to be associated with improved care processes and outcomes for patients. One of the key elements in the studies was whether or not clinicians actually used the dashboards available to them. Some QI studies have mentioned that accessing dashboards may affect their full potential. None of the included studies explored how clinicians use and integrate the information provided by the quality tools into their decision-making, and so provided few insights into why some clinicians opted not to engage with the display information.

It is important to note that the studies reviewed here were almost all conducted in high-income countries. This is similar to previous reviews evaluating Audit and Feadback (A&F) interventions; and high-income countries dominate the development of A&F interventions.^39^,^41^ For instance, a Cochrane review, which included 121 trials evaluating A&F interventions, identified just 4 trials not conducted in developed countries.^6^ As a derivative of A&F interventions, performance visualisation tools are inextricably linked to the implementation of A&F. The overall effectiveness of performance display tools may also be affected by geographical bias in evidence generation. The reasons for this geographical bias are manifold, including technical and financial constraints.^24 42^ To provide more comprehensive and generalisable evidence, future research efforts should prioritise assessments in low-income and middle-income countries and regions. Furthermore, we found that clearly distinguishing primary, secondary and tertiary care practices based solely on their definitions is difficult and not always straightforward, even when looking at a single country or region. They are often intertwined. Many diagnoses and prescriptions are often provided in multiple settings, and the settings in which these practices are delivered often vary between countries and their respective healthcare systems. Therefore, how to better localise the implementation of this intervention to exert its real influence is worthy of more exploration.

There are several limitations of this study. First, significant heterogeneity in included studies precluded the generation of an overall pooled estimate through meta-analysis. Despite the heterogeneity of outcome measurements, we consolidated outcomes of interest as RRs to enable comparison and interpretation of the effectiveness. Our inclusion criteria required that performance display be the only intervention or the core, essential feature. Isolating a single intervention is commendable in improvement science. This was necessary to avoid including multifaceted interventions where visualisation was included and where the main effects of the intervention were unlikely to be due to visualisation. One must acknowledge, however, that the exclusion of multifaceted interventions that incorporated visualisation components could lead to conservative bias (and vice versa), even though we did not estimate the effect size. We only included studies published in English, and might therefore have missed some relevant studies published in other languages. Similarly, there may be publication bias, as interventions that were unsuccessful might be less likely to have been submitted or accepted for publication. Based on the methodological limitations across included studies, this study highlights the need for more high-quality research with appropriate statistical methodology to further understand the impact in all healthcare settings. Additionally, there is a need for a larger number of RCTs and observational studies looking at the same outcome in individual departments.

Our ultimate goal is to explore ways to stimulate clinicians to provide higher quality and equal medical services to every patient. However, based on different performance data, it may have different degrees of impact on clinicians’ behaviour, but this depends more on the behavioural change mechanism. Clinicians’ decision-making processes have both fast and slow pathways, with deliberate thinking and reliance on historical patterns and well-known algorithms. It is not yet clear whether the mechanism of action of visualisation tools is ‘impact’ or the ‘reflection’ it triggers. Visualisation tools serve as downstream tools for performance data and rely on raw performance data and performance measures. Currently, most of the data used to evaluate effectiveness comes from data collection in specific settings. Using electronic health records might efficiently reduce the need for information capture and feedback delivery. Future research could look at how to increase the sustainability and intensity of this kind of intervention through clearly defined performance metrics and the incorporation of better design features, such as timeliness.

## Conclusion

We identified a small number of highly heterogeneous studies that applied performance visualisation tools as a core or separate component of strategies to quantify changes in clinician behaviour. The available evidence is insufficient to substantiate the hypothesis that this intervention has a significant effect on improving the overall quality of care via changing clinicians’ behaviour. However, it’s worth noting that visualisation of performance data is increasingly being used and researched. In this study, we focused on the broad effects of this intervention, and we noted that the intervention had mostly shown positive effects when applied across different departments, different outcome measures and different sources of performance data. This is sufficient to demonstrate the potential of this intervention. Future research could explore its specific effectiveness in a more context-specific manner, for example, to assess whether this intervention has consistent effects both as a stand-alone intervention and as part of a QI programme. Visualisation tools are becoming more widely implemented in practice and these should be accompanied by evaluations of how they improve performance as part of ongoing QI activities. Further evidence is needed to support the hypothesis that this intervention is effective, and there is a need for the development of visualisation tools that more precisely target critical areas to improve clinician performance and service quality.

## supplementary material

10.1136/bmjopen-2023-083620 online supplemental file 1

## Data Availability

Data sharing is not applicable as no data sets were generated and/or analysed for this study. All data relevant to the study are included in the article or uploaded as supplementary information.
