# Supplementary material for: Impact of visualising healthcare quality performance: a systematic review
Source: BMJ Open. 2024 Nov 2;14(11):e083620. doi: 10.1136/bmjopen-2023-083620 (PMC11535674; doi:10.1136/bmjopen-2023-083620)
Supplement: online supplemental file 1 [file bmjopen-14-11-s001.pdf]

Appendix 1 search strategy

Ovid MEDLINE(R) ALL <1946 to December 06, 2022>			Embase <1974 to 2022 December 06>		
1	(dashboard* or score?card* or white board or status board or whiteboard or electronic tracking board).tw.	3622	1	(dashboard* or score?card* or white board or status board or whiteboard or electronic tracking board).tw.	6089
2	(presentation format or display format).tw.	506	2	(presentation format or display format).tw.	615
3	(interactive plot or control panel or indicator panel).tw.	335	3	(interactive plot or control panel or indicator panel).tw.	575
4	(data display or data visuali?ation).tw.	2687	4	(data display or data visuali?ation).tw.	3237
5	exp Data Visualization/ or exp Data Display/	54826	5	exp data visualization/	2184
6	(statistical process control chart or spc chart or process control).tw.	3994	6	data display.tw.	606
7	(run chart or funnel chart or infographic or information graphic or control chart or visual display).tw.	3080	7	(statistical process control chart or spc chart or process control).tw.	5783
8	1 or 2 or 3 or 4 or 5 or 6 or 7	67764	8	(run chart or funnel chart or infographic or information graphic or control chart or visual display).tw.	4284
9	exp Health Personnel/	597824	9	1 or 2 or 3 or 4 or 5 or 6 or 7 or 8	21349
10	exp Hospitals/	310729	10	exp health care personnel/	1865797
11	exp Professional Practice/	266010	11	health care personnel.tw.	3690
12	exp Nurse Clinicians/	8492	12	exp physician/	911748
13	exp General Practitioners/	10160	13	(clinician or doctor or nurse).tw.	338863
14	exp Nurse Practitioners/	18965	14	(health care provider or healthcare provider).tw.	16889
15	(clinician or doctor or nurse).tw.	252245	15	practitioner.tw.	68265
16	(health care provider or healthcare provider).tw.	12146	16	healthcare professional.tw.	5691
17	exp Physicians/	171832	17	exp hospital/	1342125
18	practitioner.tw.	54260	18	exp professional practice/	734570
19	healthcare professional.tw.	3283	19	exp clinical nurse specialist/	2805
20	9 or 10 or 11 or 12 or 13 or 14 or 15 or 16 or 17 or 18 or 19	1285716	20	exp general practitioner/	113493
21	(health care quality or healthcare quality or quality of healthcare or quality of health care or quality of care).tw.	71604	21	exp nurse practitioner/	28970
22	quality service.tw.	811	22	exp medical specialist/	87270
23	exp "Quality of Health Care"/	7964514	23	10 or 11 or 12 or 13 or 14 or 15 or 16 or 17 or 18 or 19 or 20 or 21 or 22	3531766
24	exp Quality Assurance, Health Care/	350184	24	(health care quality or healthcare quality or quality of healthcare or quality of health care or quality of care).tw.	96996
25	(quality improvement or quality control).tw.	98291	25	quality service.tw.	1230

26	exp Quality Indicators, Health Care/	24568	26	exp health care quality/	3937319
27	exp Quality Improvement/	32830	27	exp total quality management/	84664
28	exp "Outcome and Process Assessment, Health Care"/	1342200	28	quality management.tw.	11478
29	performance.tw.	1212697	29	exp health care quality/	3937319
30	(behavi?r change or change behavi?r).tw.	12614	30	exp quality control/	493864
31	((health* personnel or health care personnel or physician? or doctor? or clinician? or nurse? or provider? or practitioner? or professional? or nursing or clinical) adj3 (skill or skills or behaviour or behavior or competence)).tw.	50774	31	performance.tw.	1442368
32	Benchmarking/	16459	32	(behavi?r change or change behavi?r).tw.	14285
33	benchmark*.tw.	57367	33	((health* personnel or health care personnel or physician? or doctor? or clinician? or nurse? or provider? or practitioner? or professional? or nursing or clinical) adj3 (skill or skills or behaviour or behavior or competence)).tw.	65535
34	Professional Competence/	25062	34	exp benchmarking/	9657
35	Clinical Competence/	103786	35	benchmark*.tw.	68872
36	Practice Patterns, Physicians'/	66512	36	professional competence/	33762
37	Practice Patterns, Nurses'/	2960	37	clinical competence/	67113
38	Practice Patterns, Dentists'/	2532	38	clinical practice/	341541
39	(practice pattern or pattern of practice or quality metric*).tw.	4789	39	nursing practice/	5889
40	exp Quality Control/	51674	40	dental practice/	1962
41	21 or 22 or 23 or 24 or 25 or 26 or 27 or 28 or 29 or 30 or 31 or 32 or 33 or 34 or 35 or 36 or 37 or 38 or 39 or 40	8969087	41	(practice pattern or pattern of practice or quality metric*).tw.	8152
42	8 and 20 and 41	2065	42	24 or 25 or 26 or 27 or 28 or 29 or 30 or 31 or 32 or 33 or 34 or 35 or 36 or 37 or 38 or 39 or 40 or 41	5870296
43	limit 42 to (abstracts and english language and humans)	1659	43	9 and 23 and 42	3656
			44	limit 43 to (abstracts and human and english language)	3251

Appendix 2 PRISMA checklist

Section and Topic	Item #	Checklist item	Location where item is reported
**TITLE**			
Title	1	Identify the report as a systematic review.	1
**ABSTRACT**			
Abstract	2	See the PRISMA 2020 for Abstracts checklist.	1
**INTRODUCTION**			
Rationale	3	Describe the rationale for the review in the context of existing knowledge.	3
Objectives	4	Provide an explicit statement of the objective(s) or question(s) the review addresses.	3
**METHODS**			
Eligibility criteria	5	Specify the inclusion and exclusion criteria for the review and how studies were grouped for the syntheses.	4
Information sources	6	Specify all databases, registers, websites, organisations, reference lists and other sources searched or consulted to identify studies. Specify the date when each source was last searched or consulted.	4
Search strategy	7	Present the full search strategies for all databases, registers and websites, including any filters and limits used.	4 and appendix1
Selection process	8	Specify the methods used to decide whether a study met the inclusion criteria of the review, including how many reviewers screened each record and each report retrieved, whether they worked independently, and if applicable, details of automation tools used in the process.	4
Data collection process	9	Specify the methods used to collect data from reports, including how many reviewers collected data from each report, whether they worked independently, any processes for obtaining or confirming data from study investigators, and if applicable, details of automation tools used in the process.	4
Data items	10a	List and define all outcomes for which data were sought. Specify whether all results that were compatible with each outcome domain in each study were sought (e.g. for all measures, time points, analyses), and if not, the methods used to decide which results to collect.	4
	10b	List and define all other variables for which data were sought (e.g. participant and intervention characteristics, funding sources). Describe any assumptions made about any missing or unclear information.	4
Study risk of bias assessment	11	Specify the methods used to assess risk of bias in the included studies, including details of the tool(s) used, how many reviewers assessed each study and whether they worked independently, and if applicable, details of automation tools used in the process.	4
Effect measures	12	Specify for each outcome the effect measure(s) (e.g. risk ratio, mean difference) used in the synthesis or presentation of results.	5
Synthesis methods	13a	Describe the processes used to decide which studies were eligible for each synthesis (e.g. tabulating the study intervention characteristics and comparing against the planned groups for each synthesis (item #5)).	5
	13b	Describe any methods required to prepare the data for presentation or synthesis, such as handling of missing summary statistics, or data conversions.	5
	13c	Describe any methods used to tabulate or visually display results of individual studies and syntheses.	5
	13d	Describe any methods used to synthesize results and provide a rationale for the choice(s). If meta-analysis was performed, describe the model(s), method(s) to identify the presence and extent of statistical heterogeneity, and software package(s) used.	NA
	13e	Describe any methods used to explore possible causes of heterogeneity among study results (e.g. subgroup analysis, meta-regression).	NA
	13f	Describe any sensitivity analyses conducted to assess robustness of the synthesized results.	NA
Reporting bias assessment	14	Describe any methods used to assess risk of bias due to missing results in a synthesis (arising from reporting biases).	4
Certainty assessment	15	Describe any methods used to assess certainty (or confidence) in the body of evidence for an outcome.	4

Section and Topic	Item #	Checklist item	Location where item is reported
**RESULTS**			
Study selection	16a	Describe the results of the search and selection process, from the number of records identified in the search to the number of studies included in the review, ideally using a flow diagram.	5 and figure 1
	16b	Cite studies that might appear to meet the inclusion criteria, but which were excluded, and explain why they were excluded.	Figure 1
Study characteristics	17	Cite each included study and present its characteristics.	5
Risk of bias in studies	18	Present assessments of risk of bias for each included study.	Appendix 5
Results of individual studies	19	For all outcomes, present, for each study: (a) summary statistics for each group (where appropriate) and (b) an effect estimate and its precision (e.g. confidence/credible interval), ideally using structured tables or plots.	6-7 and Figure 2
Results of syntheses	20a	For each synthesis, briefly summarise the characteristics and risk of bias among contributing studies.	6-7 and figure 2
	20b	Present results of all statistical syntheses conducted. If meta-analysis was done, present for each the summary estimate and its precision (e.g. confidence/credible interval) and measures of statistical heterogeneity. If comparing groups, describe the direction of the effect.	NA
	20c	Present results of all investigations of possible causes of heterogeneity among study results.	NA
	20d	Present results of all sensitivity analyses conducted to assess the robustness of the synthesized results.	NA
Reporting biases	21	Present assessments of risk of bias due to missing results (arising from reporting biases) for each synthesis assessed.	Appendix 5
Certainty of evidence	22	Present assessments of certainty (or confidence) in the body of evidence for each outcome assessed.	NA
**DISCUSSION**			
Discussion	23a	Provide a general interpretation of the results in the context of other evidence.	7-8
	23b	Discuss any limitations of the evidence included in the review.	10
	23c	Discuss any limitations of the review processes used.	10
	23d	Discuss implications of the results for practice, policy, and future research.	10
**OTHER INFORMATION**			
Registration and protocol	24a	Provide registration information for the review, including register name and registration number, or state that the review was not registered.	NA
	24b	Indicate where the review protocol can be accessed, or state that a protocol was not prepared.	NA
	24c	Describe and explain any amendments to information provided at registration or in the protocol.	NA
Support	25	Describe sources of financial or non-financial support for the review, and the role of the funders or sponsors in the review.	19
Competing interests	26	Declare any competing interests of review authors.	19
Availability of data, code and other materials	27	Report which of the following are publicly available and where they can be found: template data collection forms; data extracted from included studies; data used for all analyses; analytic code; any other materials used in the review.	19

Appendix 3 Synthesis Without Meta-analysis checklist

The citation for the Synthesis Without Meta-analysis explanation and elaboration article is: Campbell M, McKenzie JE, Sowden A, Katikireddi SV, Brennan SE, Ellis S, Hartmann-Boyce J, Ryan R, Shepperd S, Thomas J, Welch V, Thomson H. Synthesis without meta-analysis (SWiM) in systematic reviews: reporting guideline BMJ 2020;368:l6890 http://dx.doi.org/10.1136/bmj.l6890

**SWiM is intended to complement and be used as an extension to PRISMA**			
**SWiM reporting item**	**Item description**	**Page in manuscript where item is reported**	**Other***
Methods			
**1** Grouping studies for synthesis	1a) Provide a description of, and rationale for, the groups used in the synthesis (e.g., groupings of populations, interventions, outcomes, study design)	4	
	1b) Detail and provide rationale for any changes made subsequent to the protocol in the groups used in the synthesis	4	
**2** Describe the standardised metric and transformation methods used	Describe the standardised metric for each outcome. Explain why the metric(s) was chosen, and describe any methods used to transform the intervention effects, as reported in the study, to the standardised metric, citing any methodological guidance consulted	4	
**3** Describe the synthesis methods	Describe and justify the methods used to synthesise the effects for each outcome when it was not possible to undertake a meta-analysis of effect estimates	4	
**4** Criteria used to prioritise results for summary and synthesis	Where applicable, provide the criteria used, with supporting justification, to select the particular studies, or a particular study, for the main synthesis or to draw conclusions from the synthesis (e.g., based on study design, risk of bias assessments, directness in relation to the review question)	4	
**SWiM reporting item**	**Item description**	**Page in manuscript where item is reported**	**Other***
**5** Investigation of heterogeneity in reported effects	State the method(s) used to examine heterogeneity in reported effects when it was not possible to undertake a meta-analysis of effect estimates and its extensions to investigate heterogeneity	4	
**6** Certainty of evidence	Describe the methods used to assess certainty of the synthesis findings	4	
**7** Data presentation methods	Describe the graphical and tabular methods used to present the effects (e.g., tables, forest plots, harvest plots).<br>Specify key study characteristics (e.g., study design, risk of bias) used to order the studies, in the text and any tables or graphs, clearly referencing the studies included	4	
Results			
**8** Reporting results	For each comparison and outcome, provide a description of the synthesised findings, and the certainty of the findings. Describe the result in language that is consistent with the question the synthesis addresses, and indicate which studies contribute to the synthesis	5-7	
Discussion			
**9** Limitations of the synthesis	Report the limitations of the synthesis methods used and/or the groupings used in the synthesis, and how these affect the conclusions that can be drawn in relation to the original review question	10	

PRISMA=Preferred Reporting Items for Systematic Reviews and Meta-Analyses.
*If the information is not provided in the systematic review, give details of where this information is available (e.g., protocol, other published papers (provide citation details), or website (provide the URL)).

Appendix 4 Description of included studies

Study/year (ref)	Teaching hospital	Sample size of participants	Number of related patients	Real time or past performance	Performance data source	How intervention delivered	Whether introduce the intervention to clinician	Whether have reminder of intervention
Lau et al 2016 (26)	Yes	49	865	Past	EHR	Monthly Email	Yes	Yes (monthly)
Parks et al 2021 (29)	NA	63	NA	Both	EHR	Separate tool	Yes	No
Banerjee et al 2016 (22)	Yes	NA	1416	Past	EHR	Separate tool	No	No
Linder et al 2010 (27)	No	573	136633	Past	EHR	Separate tool	Yes	Yes (monthly)
Inra et al 2016 (24)	NA	28	1987	Past	EHR	Letter	No	No
Aboagye et al 2021 (21)	Yes	45	4088	Past	EHR	Separate tool with link in email	No	Yes
Kadakia et al 2020 (25)	Yes	64	81822	Past	EHR	email	Yes	No
Yan et al 2021 (23)	No	45	55364	Past	EHR	email	Yes	No
Peek et al 2020 (30)	No	43	107112	Nearly real time (daily update)	EHR	Separate tool	No	Yes
Twohig et al 2019 (31)	No	120	NA	Past	EHR	Separate tool attached in EHR	Yes	Yes (monthly)
Vikas et al 2018 (32)	Mixed	NA	12785	Past	EHR	email	No	Yes (quarterly)

Meidani et al 2021 (28)	Yes	NA	395	Past	EHR	Separate tool with link in email and color print	Yes	No

Appendix 5 Risk of bias

Study	type	D1	D2	D3	D4	D5	D6	D7	Overall
Brandyn D Lau 2016	cohort	Low	Low	Low	Low	Low	Moderate	Moderate	Moderate
Dale A Parks 2021	cohort	Moderate	Low	Moderate	Moderate	Moderate	Moderate	Moderate	Serious
Dipanjan Banerjee 2016	cohort	Moderate	Low	Moderate	Low	Moderate	Moderate	Low	Serious
Jennifer A. Inra 2016	cohort	Low	Low	Low	Low	Low	Low	Low	Low
Jonathan K. Aboagye 2021	cohort	Low	Low	Low	Low	Low	Moderate	Low	Low
Kevin Kadakia 2020	cohort	Moderate	Low	Low	Low	Low	Moderate	Low	Low
Niels Peek 2020	cohort	Moderate	Low	Low	Low	Low	Moderate	Low	Low
Patrick A. Twohig 2019	cohort	Moderate	Low	Moderate	Moderate	Moderate	Moderate	Moderate	Serious
Vikas N O'Reilly-Shah 2018	cohort	Moderate	Low	Low	Low	Low	Moderate	Low	Low
Zahra Meidani 2021	cohort	Moderate	Low	Low	Moderate	Low	Moderate	Low	Moderate
Jeffrey A Linder 2010	RCT	Some concern	Low	Low	Some concern	Low			Some concern
Lily Du Yan 2021	RCT	Low	Low	Low	Some concern	Some concern			Some concern

Appendix 6 Forest plot of performance change outcome in relative risk

Study	Outcome	Outcome group	Intervention Events	Total	Comparator Events	Total	RR	95%CI
Brandyn D Lau 2016	Prescribing Risk–appropriate VTE Prophylaxis	Prescribing	748	784	773	865	1.07	(1.04, 1.1)
Dale A Parks 2021	Adherence with clinical guidelines	Prescribing	3143	3716	4056	6103	1.27	(1.24, 1.3)
Dipanjan Banerjee 2016	Heart failure 30–day readmission	Monitoring	40	397	67	480	1.39	(1.02, 1.75)
Jeffrey A Linder 2010	Antibiotic prescribing for ARIs	Prescribing	3912	8406	4761	10082	1.01	(0.98, 1.05)
Jennifer A. Inra 2016	Adenoma Detection for male ... 25%	Use of laboratory tests	325	1083	306	959	0.94	(0.83, 1.07)
Jonathan K. Aboagye 2021	Surgical patients prescribed risk–appropriate VTE prophylaxis	Prescribing	806	819	421	440	1.03	(1.01, 1.05)
Kevin Kadakia 2020	Utilization of CT	Use of laboratory tests	11437	45726	12032	36096	1.33	(1.31, 1.35)
Lily Du Yan 2021	Antibiotic prescription in URI	Prescribing	487	6247	884	6907	1.64	(1.54, 1.75)
Lily Du Yan 2021	Antibiotic prescription in bronchitis	Prescribing	757	2357	1058	2997	1.1	(1.02, 1.18)
Lily Du Yan 2021	Antibiotic prescription in sinusitis	Prescribing	8730	11367	11013	14359	1	(0.99, 1.01)
Lily Du Yan 2021	Antibiotic prescription in pharyngitis	Prescribing	3630	5542	5396	7166	1.15	(1.13, 1.17)
Niels Peek 2020	Receipt of 1 or more potentially hazardous prescriptions	Prescribing	778	47163	1230	47183	1.58	(1.49, 1.67)
Niels Peek 2020	Inadequate prescription monitoring	Monitoring	61	652	89	668	1.42	(1.12, 1.73)
Patrick A. Twohig 2019	Colorectal cancer screening	Use of laboratory tests	31576	53671	27491	50515	1.08	(1.07, 1.09)
Patrick A. Twohig 2019	Patients with diabetes who had an A1c greater than 9% or no A1c in the past year	Monitoring	6201	18446	6100	17390	1.04	(1.01, 1.07)
Vikas N O'Reilly–Shah 2018	Compliance with VT8 ventilation	Prescribing	580	888	3150	5309	1.1	(1.04, 1.16)
Zahra Meidani 2021	Duplicated test	Use of laboratory tests	12	167	31	228	1.89	(1.26, 2.53)

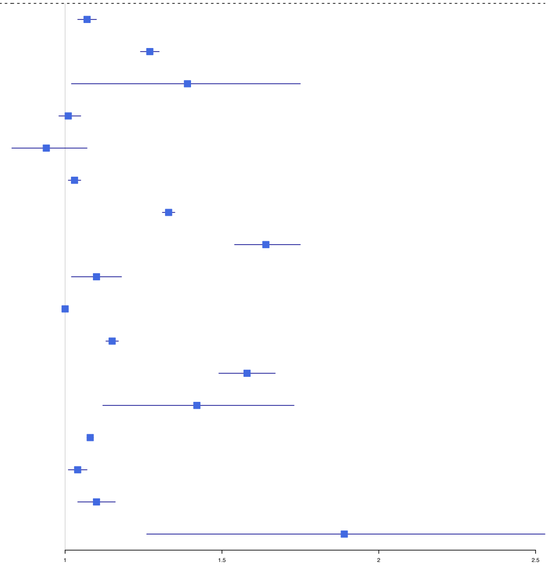

Abbreviations

VTE: Venous thromboembolism; ARIs: Acute respiratory infections; CT: computed tomography scan; URI: Urinary tract infections; A1c: glycated hemoglobin; VT8: tidal volumes <8mL/kg
