# Supplementary material for: Impact of visualising healthcare quality performance: a systematic review
Source: BMJ Open. 2024 Nov 2;14(11):e083620. doi: 10.1136/bmjopen-2023-083620 (PMC11535674; doi:10.1136/bmjopen-2023-083620)
Supplement: Supplementary file 2 [file bmjopen-2023-083620.reviewer_comments.pdf]

PEER REVIEW HISTORY

BMJ Open publishes all reviews undertaken for accepted manuscripts. Reviewers are asked to complete a checklist review form and are provided with free text boxes to elaborate on their assessment. These free text comments are reproduced below.

ARTICLE DETAILS

Title (Provisional)

Impact of visualizing health care quality performance: a systematic review

Authors

Yang, Zijing; Alveyn, Edward; Dey, Mrinalini; Arumalla, Nikita; Russell, Mark D; Norton, Sam; Galloway, James B

VERSION 1 - REVIEW

**Reviewer**	**1**
**Name**	**Gagnon, Justin**
**Affiliation**	**Université de Sherbrooke**
**Date**	**06-Feb-2024**
**COI**	**I have no competing interests to declare.**

The article "Impact of visualizing health care quality performance: a systematic review" examines the effectiveness of health data visualization tools to enhance care processes and outcomes. It synthesizes evidence from various studies to assess how visual tools like dashboards and scorecards impact healthcare professional performance and, consequently, healthcare quality.

Abstract

The abstract is succinctly establishes the study’s scope and objectives, however I feel that the background could better detail the need to synthesize research on this topic – why visualization interventions are important, and what will conducting a broader synthesis that isn’t department-specific provide?

In the methods, I suggest providing additional detail and rationale regarding the selection criteria, the study designs you focused on, what data were extracted, and how you established what relevant data would be extracted.

The results section provides a rather superficial overview of the characteristics of the studies included in the review, and whether or not significant change was observed. However, I would suggest providing additional detail that explains the differences observed (the key

factors that could influence the effectiveness or explain variation). I would also suggest alluding to the implications of your findings for research and/or practice.

Introduction

I feel that your introduction lacks a comprehensive exploration of the nature and scope of the problem related to visualizing healthcare quality performance. It begins with a general overview of quality improvement (QI) programs, and then abruptly moves onto a discussion of the shift towards digital and visual tools in healthcare. In addition, the need for this systematic review is stated, but the introduction does not effectively argue why previous reviews are insufficient or how this review will add to the existing body of knowledge. A more critical assessment of existing literature and a clearer articulation of the unique contribution of this review would strengthen this section.

I might be misinterpreting, but this sentence in your introduction seems to say something different than the first sentence of your abstract.

This current update aims to investigate the effectiveness of visualization interventions on improving care processes and outcomes, with a focus only on individual-specific or department-specific performance display tools.

Current reviews which aim to investigate the effectiveness of visualization interventions on improving care

processes and outcomes only focus on department-specific performance change.

Methods

The methodology section could benefit from more detailed explanation of the search strategy and selection criteria. For instance, are the logical operators between "dashboard", "data displays" … and "clinician" OR or AND (I see that it's synonyms of data visualization AND synonyms of clinician…)? What was the date range for the studies searched, and why those dates? What language? Did you follow a process for identifying the search terms you selected, that might convey methodological rigor? What process did you follow for identifying eligible articles? According to what characteristics were you able to eliminate ineligible studies (study design, nature of the intervention, etc.) at the title/abstract review stage, and then at the full-text review stage?

The authors appear to have followed a rigorous approach to data extraction, involving independent review by multiple researchers and consensus discussion. I recommend additional detail regarding the data extraction process, however. Specifically, what process did you follow to develop an extraction grid?

In the data synthesis section, I suggest justifying why a narrative approach was chosen.

Heterogeneity and Limitations: The acknowledgement of heterogeneity in study designs and outcomes is important. The authors' approach to assess heterogeneity qualitatively is

appropriate given the circumstances, but it might not provide a robust measure of variability between studies.

Results

The results provide adequate detail about the intervention characteristics, outcome measures, and effectiveness of interventions. I feel, however, that additional detail should be provided in the results section that addresses your third objective. I see that the discussion addresses this third objective, but I feel that at least a foreshadowing of this discussion should be presented in the results section. Perhaps a more explicit presentation of the qualitative data extraction fields in the methods section would give the reader a better sense of what characteristics and factors you sought to compare (including the nature of the interventions, types of settings, nature of the studies, outcome measures, etc.), to explain the variable outcomes.

Discussion

I commend the authors on their being forthcoming about how the information provided within the articles did not meet their expectations and limited their capacity to make in depth comparisons, and for recommendation caution when interpreting the results. Likewise, that the heterogeneity of outcome measurements (as well as heterogeneity of settings and types of interventions) limited their capacity to perform meta-analysis.

I feel that strengthening the qualitative comparison component of your article would provide additional insight into where and how and what kinds of visual interventions are used, help you overcome the above limitations, and thus greatly improve the value of your research.

**Reviewer**	**2**
**Name**	**de la Perrelle, Lenore**
**Affiliation**	**Flinders University, of Education, Psychology and Social Work**
**Date**	**25-Feb-2024**
**COI**	**Nil.**

This is an interesting review of the use of dashboards in monitoring improvement or behaviour change of health professionals. It is well written and conforms to systematic review requirements.

I would like to see references to the way reverse scored data was converted at line 7 P.6 and other references to the statistical procedures used. This would ensure that the review could be replicated using the same techniques.

There may be a typographical error on line 32, P.9 to be corrected.

I would have liked some discussion about the existence of any qualitative data suggesting how the dashboards or A&F may have influenced changed behaviour.

In some QI papers, there will be some data about how this reminded or prompted behaviour change. This may be an area for further exploration about the effectiveness of dashboards in changing health professionals behaviour.

**Reviewer**	**3**
**Name**	**Knudsen, Søren**
**Affiliation**	**Aalborg University, Danish Center for Health Services Research**
**Date**	**25-Feb-2024**
**COI**	**None.**

Thanks to the authors for an interesting and well-written article. It presents a valuable study within the field of Improvement Science, which lacks extensive scientific evaluations of improvement processes. There are some revisions that need to be addressed. However, these revisions are relatively minor and should not be insurmountable.

**

Reviewers' comments

This systematic review includes studies assessing the effects of data visualization tools as a single or core intervention on care processes and outcomes. The outcomes of interest were consolidated as relative risks (RRs) to enable comparison, and a narrative approach was used to describe the evidence relating to the chosen outcome measures. The review identified 12

relevant studies, comprising two randomized controlled trials (RCTs) and ten observational studies.

Overall, this is an interesting and well-written article. It presents a valuable study within the field of improvement science, which lacks extensive scientific evaluations of improvement processes. However, there are several revisions that need to be addressed. However, these revisions are relatively minor and should not be insurmountable:

Abstract:

Lines 9-13: The background section is unclear. It does not articulate the difference between existing reviews and this one.

Lines 22-23: "Intervention, that reported quantitative clinical behaviour changes were included." The term 'clinical behaviour changes' is undefined anywhere in the document.

Introduction:

Generally: The discussion indicates the presence of several systematic reviews on the use of data visualization tools. This should be clarified in the introduction regarding what already exists and how this article differs from existing reviews.

Line 40: "This current update aims" is vague. What is meant by 'current update'? An update of what?

Lines 42-44: "...with a focus only on individual-specific (14) or department-specific (15) performance display tools" needs clarification on its meaning and relevance, and how it differs from other reviews.

Methods:

Generally: Mention the use of the PRISMA statement for reporting.

Was a protocol established in advance? If so, mention this according to PRISMA guidelines. If not, this should be noted under limitations.

Lines 26-27: Are quality improvement projects, such as PDSA-based projects, excluded?

Results:

Page 6, Line 32: "We used a pragmatic classification method to group studies with similar outcomes (Figure 2)." The classifications are not evident in Figure 2.

Pages 6-7, Lines 50-32: It is mentioned that outcomes are classified as "prescribing, use of laboratory tests, or monitoring". Use this terminology consistently in this section and in the discussion section to clarify the categories.

The results from some articles are omitted. Given there are only 12 articles, consider presenting key findings from all.

Discussion:

Page 7, Line 40: The term "healthcare providers' behaviour" is frequently used. However, the focus is on the effect on process and outcome indicators, which might be a result of changed clinician behaviour. This should be elaborated in the introduction if it's a criterion for inclusion.

Pages 7-8, Lines 58-5: The strength of your review is in isolating a single intervention, which is commendable in Improvement Science. The concern about underestimating the true power of the intervention by excluding multi-component interventions is addressed by your methodology. This should be highlighted as a strength.

Page 8, Lines 17-18: "This study demonstrates a growing literature supporting the likely effectiveness of these tools" requires a reference.

Page 8, Line 46: "The previous study" is unclear. If it's the first mention of Reference 33, it should be "A previous study".

Page 9, Lines 30-33: "The overall effectiveness of performance display tools may also be affected by geographical bias in evidence generation" could benefit from a reference.

Conclusion:

Avoid using informal language such as "huge", "this is enough to", and "In short".

Table 1:
Include the category in which each study was placed.

Figure 2:
Improve the resolution. Clarify what is meant by 'intervention and control events' in the methods section. 'Control events' is misspelled. Please explain abbreviations used in the figure caption.

Other:
Write out numbers under ten in text.

**Reviewer**	**4**
**Name**	**Boggan, Joel**
**Affiliation**	**Durham Veterans Affairs Medical Center**
**Date**	**12-Mar-2024**
**COI**	**n/a**

Yang et al. performed a systematic review of visualization strategies in healthcare. They identified clinical dashboards and scorecards as primary interventions within the 12 included studies, but little is shared about how these interventions were actually performed at the individual study level. Including more of these details would be important for the generalizability of this work given the ubiquity of these visualizations in modern healthcare and the very broad general objectives. A previous study on dashboards was mentioned in the references but seems to have included some studies that were not included here although the topics were somewhat aligned. Emphasizing the differences in these two studies might provide some important structure to the background and discussion that may improve clarity.

MAJOR ISSUES

-Reference 31 is specifically on clinical dashboards and is mentioned in the discussion. Given that eight of their studies looked at dashboards as standalone interventions, it seems this might be important background information for your introduction and part of the justification for the current study may be how your study was intended to differ from this one, given they aimed to ‘assess the effectiveness of clinical dashboards used as CDSS or

A&F tools (as either a standalone intervention or part of a multifaceted intervention). Also, why was only one of their 8 studies of dashboards as a standalone intervention included here?

-Additionally, given this review exists, I don't think you can claim the first review of performance visualization interventions as a strength in your abstract/key points.

-I'm not sure objective 3 was ever addressed in the methods/results and probably should be eliminated from the current objective set. Additionally, there is little around what these key factors might be in the discussion.

-I think the inclusion of a forest plot may be unwarranted, given the heterogeneity in how the visualizations were discussed as being used and that you have included multiple different outcomes from the same study within the same forest plot. Even if you limited a figure to the separate studies on antibiotics for URIs or colorectal cancer screening, a forest plot with only two data points should still probably not be included. If removing the Figure, the final paragraph of the methods should probably end with the sentence beginning 'A narrative approach…' (p.5, line 11-12).

-Amongst the characteristics of these studies that would be important for readers to know would be how the studies intended the visual to be used. Was it presented at meetings, was it passively integrated into the EHR, how did clinicians know where to find it, did the clinicians actually use it (which is what I think you were discussing in p.6, lines 41-42, but is unclear to me upon re-reading)? This latter point is also mentioned in the discussion but seems like it would be important to include as a result.

-In the discussion, there is mention about grouping into the 3 strategies (p. 8, lines 31-32), which seems to refer to a single sentence in the Results (p. 6, lines 31-38). If you feel these truly are important pragmatic groupings, perhaps the discussion should be expanded or refocused to include how you think these groupings might (or might not) be distinct from how or why a visualization might be effective and/or integrated into clinical care or operations.

MINOR ISSUES

-As visualizations are the main focus of this paper, the current opening introductory paragraph is only loosely tied to the topic. If the thread to connect to the remainder of the paper is around visuals being part of audit and feedback strategies (as part of your discussion also suggests), perhaps that can somehow be integrated into the current second paragraph.

-The Methods would be improved with a stricter adherence to the PRISMA guidelines. For example, the dates of searches should be included, whether the reviews were performed in duplicate, etc.

-Was there any protocol registration and/or IRB approval for this study?

-Figure 1 should probably include separate exclusions at the title/abstract and the full text levels.

VERSION 1 - AUTHOR RESPONSE

Reviewers' comments

This systematic review includes studies assessing the effects of data visualization tools as a single or core intervention on care processes and outcomes. The outcomes of interest were consolidated as relative risks (RRs) to enable comparison, and a narrative approach was used to describe the evidence relating to the chosen outcome measures. The review identified 12 relevant studies, comprising two randomized controlled trials (RCTs) and ten observational studies.

Overall, this is an interesting and well-written article. It presents a valuable study within the field of improvement science, which lacks extensive scientific evaluations of improvement processes. However, there are several revisions that need to be addressed. However, these revisions are relatively minor and should not be insurmountable:

We would like to extend our sincere gratitude to the esteemed reviewers for their invaluable time and feedback on our manuscript. These comments and suggestions have enriched the quality of our work. We truly appreciate it. Here is the reply to each comment.

Abstract:
Lines 9-13: The background section is unclear. It does not articulate the difference between existing reviews and this one.

We have clarified this in the text stating "A Cochrane systematic literature review of 140 studies, published in 2012, found that audit and feedback can lead to small but potentially important improvements in professional practice and clinical outcomes (6). Audit and feedback may be more effective when given both verbally and in writing." "Current reviews which aim to investigate the effectiveness of visualisation interventions on improving care processes and outcomes only focus on department-specific performance change instead of in general."

Lines 22-23: "Intervention, that reported quantitative clinical behaviour changes were included." The term 'clinical behaviour changes' is undefined anywhere in the document.

We have clarified this in the text stating "MEDLINE and Embase were searched until December 2022. Randomised controlled trials (RCTs) and observational studies involving visualisation intervention, either alone or as a core intervention, that reported quantitative outcomes including process and outcome indicators were included. "

Introduction:
Generally: The discussion indicates the presence of several systematic reviews on the use of data visualization tools. This should be clarified in the introduction regarding what already exists and how this article differs from existing reviews.

Line 40: "This current update aims" is vague. What is meant by 'current update'? An update of what? Lines 42-44: "...with a focus only on individual-specific (14) or department-specific (15)

performance display tools" needs clarification on its meaning and relevance, and how it differs from other reviews.

We have clarified this in the text stating "The existing literature on visual and digital intervention primarily focuses on the development process and usability, such as more efficient comprehension of complex information and technical design of additional functions(10, 12, 13). Reviews which aim to investigate the effectiveness of visualisation interventions on improving care processes and outcomes have a focus only on individual-specific, like scorecard-specific or dashboard-specific (14, 15) or department-specific, like primary and aged care (16, 17), performance display tools. Therefore, we conducted a systematic literature review to appraise the evidence for the effectiveness of interventions involving all visualization tools of clinician performance display intended to improve process and outcome care (e.g. prescription), with the following objectives:"

Methods:
Generally: Mention the use of the PRISMA statement for reporting.

Was a protocol established in advance? If so, mention this according to PRISMA guidelines. If not, this should be noted under limitations.

We have clarified this in the text stating "The preferred reporting system for systematic reviews (PRISMA) was followed in conducting this review (16)."

Lines 26-27: Are quality improvement projects, such as PDSA-based projects, excluded?

We did not consider whether articles applied the PDSA method as an inclusion or exclusion criterion. Our inclusion and exclusion criteria all followed those mentioned in Methods. Coincidentally, among the finally included articles, no article contained the term PDSA.

Results:
Page 6, Line 32: "We used a pragmatic classification method to group studies with similar outcomes (Figure 2)." The classifications are not evident in Figure 2.

Have updated.

Pages 6-7, Lines 50-32: It is mentioned that outcomes are classified as "prescribing, use of laboratory tests, or monitoring". Use this terminology consistently in this section and in the discussion section to clarify the categories.

Have checked.

The results from some articles are omitted. Given there are only 12 articles, consider presenting key findings from all.

Discussion:
Page 7, Line 40: The term "healthcare providers' behaviour" is frequently used. However, the focus is on the effect on process and outcome indicators, which might be a result of changed clinician behaviour. This should be elaborated in the introduction if it's a criterion for inclusion.

We thank the reviewer for highlighting these important points about the clear definition. It is not an inclusion criterion. In the final extracted outcome measures, some outcomes where the actor is not a clinician have also been included. In this study, we are discussing the effectiveness of this intervention for clinicians from the perspective of the service they provide.

Have clarified "The included studies were required to directly report, or provide sufficient information to derive, the following data elements necessary for standardisation: (1) sample size, and (2) quantitative outcome measurement including process and outcome indicators."

Pages 7-8, Lines 58-5: The strength of your review is in isolating a single intervention, which is commendable in Improvement Science. The concern about underestimating the true power of the intervention by excluding multi-component interventions is addressed by your methodology. This should be highlighted as a strength.

Page 8, Lines 17-18: "This study demonstrates a growing literature supporting the likely effectiveness of these tools" requires a reference.

We have clarified this in the text stating "This study demonstrates a growing literature supporting the likely effectiveness of these tools as half of the included studies in our review are published in the last 3 years. "

Page 8, Line 46: "The previous study" is unclear. If it's the first mention of Reference 33, it should be "A previous study".

Thanks for your kind comment. Have changed

Page 9, Lines 30-33: "The overall effectiveness of performance display tools may also be affected by geographical bias in evidence generation" could benefit from a reference.

Thanks for your kind comment. Have added

Conclusion:
Avoid using informal language such as "huge", "this is enough to", and "In short".

Thanks for your kind comment. Have changed

Table 1:
Include the category in which each study was placed.

We have categorised included studies by country, study design, placed setting and the intervention type.

Figure 2:
Improve the resolution. Clarify what is meant by 'intervention and control events' in the methods section. 'Control events' is misspelled. Please explain abbreviations used in the figure caption.

As mentioned in method:

"As such, we only included studies that evaluated a visualisation intervention as a single or core intervention."

"Potential comparators considered were the pre-intervention baseline, no intervention, or any other intervention (single or multifaceted) not involving performance data display."

Other:
Write out numbers under ten in text.

Thanks for your kind comment. Have changed.

VERSION 2 - REVIEW

**Reviewer**	**2**
**Name**	**de la Perrelle, Lenore**
**Affiliation**	**Flinders University, of Education, Psychology and Social Work**
**Date**	**16-Apr-2024**
**COI**	**None**

Thank you for reviewing the manuscript. It appears that not all comments are responded to in comments.

The revised manuscript would benefit from a few minor terminology and wording changes to clarify further.

Abstract:L12 'in general' is still not clear. Consider rephrasing to 'overall effectiveness"

L14 would be useful to add 'this review addresses the gap in evidence for the overall effectiveness of visual display performance interventions'

Introduction

p 5 L46-50 rephrase the descriptions to avoid repeated use of like. Consider 'focus on type (scorecard or dashboard) or setting (primary health or aged care).

Specify the gap in literature: To address the gap in literature about the effects of ... display tools on ... we conducted...'

Methods

p6 L9-16 clarify dates and if a protocol was used. Short sentences may be better linked

Discussion p9 L56 indicates studies published 2020-2021, not 3 years as noted in comments responding to reviewer. need to confirm

p10 L9-22 a reflection on the value of a mixed methods review to capture additional components of an intervention may be useful and included in recommendations for further research. Many QI and behaviour change interventions would identify reasoning of clinicians in whether to engage in the intervention or why.

p12 L42-53 this section is not clear and needs a review and a reference. is it better placed in the discussion earlier than in limitations?

does this mean that the effectiveness measures alone do not indicate the mechanism of change and that exploration of this is needed?

**Reviewer**	**3**
**Name**	**Knudsen, Søren**
**Affiliation**	**Aalborg University, Danish Center for Health Services Research**
**Date**	**30-Apr-2024**
**COI**	**none**

Thank you for sending the revised edition. I appreciate the improvements made; however, there are still some areas that require attention before publication. Primarily, the introduction needs further revision to clarify the review's objectives clearly.

Abstract:

Please rewrite the background section to succinctly explain the relevance of the team, identify gaps in existing knowledge, and outline how this review aims to fill these gaps.

Introduction:

The introduction requires a thorough rewrite to clarify the purpose of the review. The text between lines 3 and 10 is particularly vague. It is essential to explain why the chosen theme is relevant, what existing knowledge covers, what gaps remain, and how this paper will address these gaps. Moreover, the link between Quality Improvement (QI) and clinicians' behavior, as mentioned in line 3, needs to be clarified. The role of audit and feedback in this context is also unclear. Additionally, if the three objectives listed at the end of the introduction are to be retained, they must be directly addressed in the discussion section or else removed.

Specific textual revisions:

On Page 6, line 11, please adjust to past tense.

On Page 7, lines 9-16, cite the relevant references in the “risk of bias” section.

On Page 7, lines 54-59 ("it is noteworthy…"), the text feels overly discursive. Consider moving this part to the discussion section or revising it to present the results more neutrally.

**Reviewer**	**4**
**Name**	**Boggan, Joel**
**Affiliation**	**Durham Veterans Affairs Medical Center**
**Date**	**30-Apr-2024**
**COI**	**[None indicated].**

[NOTE FROM THE EDITOR: The reviewer has declined to submit a re-review at this time because the authors have not responded to any of their previous comments]

VERSION 2 - AUTHOR RESPONSE

ORIGINAL REVIEWER COMMENTS FROM REVIEWERS 1, 2, AND 4 (NOT PREVIOUSLY ADDRESSED):

Reviewer: 1
Dr. Justin Gagnon, Université de Sherbrooke
Comments to the Author:
The article "Impact of visualizing health care quality performance: a systematic review" examines the effectiveness of health data visualization tools to enhance care processes and outcomes. It synthesizes evidence from various studies to assess how visual tools like dashboards and scorecards impact healthcare professional performance and, consequently, healthcare quality.
Thank you for taking the time to review our manuscript and provide valuable feedback. It seems that our previous response did not address all comments. When I downloaded the decision letter from the submission system, I could only see some of the reviewers' comments in the letter for unknown reasons. It seems that there was an issue that caused your valuable feedback to not be included in the file I received. I apologize for any inconvenience this may have caused. We have considered all your insightful suggestions and comments and made necessary revisions to improve the quality of our manuscript. Thank you for your understanding and patience. We greatly appreciate your contribution to enhancing our work.

Abstract
The abstract is succinctly establishes the study's scope and objectives, however I feel that the background could better detail the need to synthesize research on this topic – why visualization interventions are important, and what will conducting a broader synthesis that isn't department-specific provide?
In the methods, I suggest providing additional detail and rationale regarding the selection criteria, the study designs you focused on, what data were extracted, and how you established what relevant data would be extracted.
The results section provides a rather superficial overview of the characteristics of the studies included in the review, and whether or not significant change was observed. However, I would suggest providing additional detail that explains the differences observed (the key factors that could influence the effectiveness or explain variation).
I would also suggest alluding to the implications of your findings for research and/or practice.
Thank you for your comment, which is really helpful. We have made changes considering your comment.
Introduction

I feel that your introduction lacks a comprehensive exploration of the nature and scope of the problem related to visualizing healthcare quality performance. It begins with a general overview of quality improvement (QI) programs, and then abruptly moves onto a discussion of the shift towards digital and visual tools in healthcare. In addition, the need for this systematic review is stated, but the introduction does not effectively argue why previous reviews are insufficient or how this review will add to the existing body of knowledge. A more critical assessment of existing literature and a clearer articulation of the unique contribution of this review would strengthen this section.
I might be misinterpreting, but this sentence in your introduction seems to say something different than the first sentence of your abstract.
This current update aims to investigate the effectiveness of visualization interventions on improving care processes and outcomes, with a focus only on individual-specific or department-specific performance display tools.

Current reviews which aim to investigate the effectiveness of visualization interventions on improving care processes and outcomes only focus on department-specific performance change.

Thank you for your insightful feedback on our manuscript. We appreciate your comments and have taken them into careful consideration. We have revised the introduction section to clearly articulate the research gap.

Methods
The methodology section could benefit from more detailed explanation of the search strategy and selection criteria. For instance, are the logical operators between "dashboard", "data displays" … and "clinician" OR or AND (I see that it's synonyms of data visualization AND synonyms of clinician…)? What was the date range for the studies searched, and why those dates? What language? Did you follow a process for identifying the search terms you selected, that might convey methodological rigor? What process did you follow for identifying eligible articles? According to what characteristics were you able to eliminate ineligible studies (study design, nature of the intervention, etc.) at the title/abstract review stage, and then at the full-text review stage?

Thanks for your comment. The detail of the search strategy is described in the supplementary material, and the study selection paragraph has already explained these details.

The authors appear to have followed a rigorous approach to data extraction, involving independent review by multiple researchers and consensus discussion. I recommend additional detail regarding the data extraction process, however. Specifically, what process did you follow to develop an extraction grid?
Thanks for your comment. The data extraction procedure was discussed and further standardized by the research group.

In the data synthesis section, I suggest justifying why a narrative approach was chosen.
Thanks for your comment. As we explained in the method " A narrative approach was used to describe the evidence relating to the chosen outcome measures based on the direction and size of reported effects in accordance with Cochrane recommendations."

Heterogeneity and Limitations: The acknowledgement of heterogeneity in study designs and outcomes is important. The authors' approach to assess heterogeneity qualitatively is appropriate given the circumstances, but it might not provide a robust measure of variability between studies.
Thanks for your comments. Unfortunately, we are unable to quantitatively and reliably measure inter-institutional variability currently, due to the heterogeneity of methods and outcome measures. Thus, we present

a visual summary of current research and a narrative synthesis to explore the practical applications of current visualization interventions in this study.

Results
The results provide adequate detail about the intervention characteristics, outcome measures, and effectiveness of interventions. I feel, however, that additional detail should be provided in the results section that addresses your third objective. I see that the discussion addresses this third objective, but I feel that at least a foreshadowing of this discussion should be presented in the results section. Perhaps a more explicit presentation of the qualitative data extraction fields in the methods section would give the reader a better sense of what characteristics and factors you sought to compare (including the nature of the interventions, types of settings, nature of the studies, outcome measures, etc.), to explain the variable outcomes.
Thanks for your comment. In the method section, we mentioned that “Data were extracted on the author, country, year of publication, study aims, study design, intervention, implementation strategy, setting, population, sample size, duration, outcome measures, and results.” And the qualitative data was summarized in the baseline table. In the “*Intervention characteristics*” paragraph in the result section, we also described these factors. In the appendix, we also summarize some qualitative data including whether the intervention tool was introduced to doctors and how doctors could access it, such as directly attached to an email or integrated into the EHR.

Discussion
I commend the authors on their being forthcoming about how the information provided within the articles did not meet their expectations and limited their capacity to make in depth comparisons, and for recommendation caution when interpreting the results. Likewise, that the heterogeneity of outcome measurements (as well as heterogeneity of settings and types of interventions) limited their capacity to perform meta-analysis.
I feel that strengthening the qualitative comparison component of your article would provide additional insight into where and how and what kinds of visual interventions are used, help you overcome the above limitations, and thus greatly improve the value of your research.
Thank you for your thoughtful feedback and understanding of the limitations we faced in our study. We appreciate your compliments on the limitations of the information we provided in the article and the transparency regarding the heterogeneity of outcome measures, settings, and intervention types. Your suggestions on the need for caution in interpreting the results were very helpful. We agree that strengthening the qualitative comparison section is important and could indeed provide deeper insights and overcome some of the limitations we faced. We will consider this more in future research efforts to enhance their depth and value. Thank you again for your constructive comments and support.

Reviewer: 2
Dr. Lenore de la Perrelle, Flinders University, Flinders University
Comments to the Author:
This is an interesting review of the use of dashboards in monitoring improvement or behaviour change of health professionals. It is well written and conforms to systematic review requirements.

I would like to see references to the way reverse scored data was converted at line 7 P.6 and other references to the statistical procedures used. This would ensure that the review could be replicated using the same techniques.

Thanks for your comment. We have explained in more detail in the revised version how the relative risks were calculated.

There may be a typographical error on line 32, P.9 to be corrected.

Thanks and we have changed.

I would have liked some discussion about the existence of any qualitative data suggesting how the dashboards or A&F may have influenced changed behaviour.
In some QI papers, there will be some data about how this reminded or prompted behaviour change. This may be an area for further exploration about the effectiveness of dashboards in changing health professionals behaviour.

In some qualitative studies for developing better tools discussed how and why these tools influence clinicians' behaviors like whether the dashboards acted as reminders, prompted critical reflection on practice, or facilitated team discussions, all of which could lead to behavior changes. Unfortunately, the studies included in our review provided limited qualitative information, with only one article describing clinicians' access to the dashboard. This limited our ability to explore how the visualizations might have prompted behavior changes. We look forward to future studies that combine quantitative and qualitative data to capture these subtle aspects of behavior change. This would not only enhance our understanding of the effectiveness of the dashboards, but also help generate more reliable and actionable findings. Thank you again for your valuable feedback.

Reviewer: 4
Dr. Joel Boggan, Durham Veterans Affairs Medical Center
Comments to the Author:
Yang et al. performed a systematic review of visualization strategies in healthcare. They identified clinical dashboards and scorecards as primary interventions within the 12 included studies, but little is shared about how these interventions were actually performed at the individual study level. Including more of these details would be important for the generalizability of this work given the ubiquity of these visualizations in modern healthcare and the very broad general objectives. A previous study on dashboards was mentioned in the references but seems to have included some studies that were not included here although the topics were somewhat aligned. Emphasizing the differences in these two studies might provide some important structure to the background and discussion that may improve clarity.

MAJOR ISSUES

-Reference 31 is specifically on clinical dashboards and is mentioned in the discussion. Given that eight of their studies looked at dashboards as standalone interventions, it seems this might be important background information for your introduction and part of the justification for the current study may be how your study was intended to differ from this one, given they aimed to ‘assess the effectiveness of clinical dashboards used as CDSS or A&F tools (as either a standalone intervention or part of a multifaceted intervention). Also, why was only one of their 8 studies of dashboards as a standalone intervention included here?

Thank you for your careful review and insightful comments. We did review the articles in this review and found that several of them did not meet our inclusion criteria for this review. Specifically, some studies focused on patient exam data monitoring rather than clinician performance visualization. Other studies included visualization as part of a multifaceted intervention rather than as the core or sole intervention. In addition, some studies lacked the quantitative data required for our analysis. Therefore, we only included studies that met our criteria, focused on visualization used as the primary or core intervention, and had relevant quantitative outcomes.

-Additionally, given this review exists, I don’t think you can claim the first review of performance visualization interventions as a strength in your abstract/key points.

Thank you for your comment. Regarding the claim that our review was "first" in terms of abstract and key points, we acknowledge that there are other reviews, including the one you mentioned. However, our review focuses on a wide range of visualization tools in clinical departments and attempts to quantitatively evaluate their impact on clinician performance and care processes. While previous reviews may have focused on specific types of visualizations or specific departments, our review aims to fill this gap by providing a comprehensive evaluation of all visualization tools designed to improve performance in different healthcare settings. We apologize for the confusion. We have revised the claim. We now accurately describe the novelty of our review in terms of its comprehensive scope and the broad inclusion of various visualization tools, rather than claiming it as the first review.

-I'm not sure objective 3 was ever addressed in the methods/results and probably should be eliminated from the current objective set. Additionally, there is little around what these key factors might be in the discussion.
Thank you for pointing out the issues regarding Aim 3. After careful consideration, We agree that Aim 3 was not adequately addressed in the Methods and Results sections. Initially, we anticipated identifying and analyzing factors that could be associated with the effects of the visualization intervention. However, the studies that were ultimately included did not provide sufficient detail or consistency to allow for a meaningful synthesis of these factors. Despite our efforts to extract relevant information, the variability and quality of the data made it challenging to effectively summarize and analyze these aspects. Given this, we removed Aim 3 from the final objectives in the revised manuscript. We also revised the Discussion to clarify why these key factors could not be effectively explored and to acknowledge the limitations of the existing data. Thank you again for your insightful feedback.

-I think the inclusion of a forest plot may be unwarranted, given the heterogeneity in how the visualizations were discussed as being used and that you have included multiple different outcomes from the same study within the same forest plot. Even if you limited a figure to the separate studies on antibiotics for URIs or colorectal cancer screening, a forest plot with only two data points should still probably not be included. If removing the Figure, the final paragraph of the methods should probably end with the sentence beginning 'A narrative approach…' (p.5, line 11-12).
Thank you for your detailed review and your feedback on the use of forest plots. I would like to clarify the purpose and application of forest plots in our study. The forest plots in our manuscript are not intended for meta-analysis or data aggregation, but rather to visually summarize the range of results reported across studies.
This plot was included to illustrate the variation in effect sizes and the direction of the observed effects across studies, rather than to provide summary estimates. This approach facilitates understanding of the dispersion of results and provides context for the narrative synthesis of our findings.
We acknowledge that forest plots are often used in meta-analyses with a sufficient number of studies, but in this case, it was used to provide a clear, visual representation of the results of the included studies.

-Amongst the characteristics of these studies that would be important for readers to know would be how the studies intended the visual to be used. Was it presented at meetings, was it passively integrated into the EHR, how did clinicians know where to find it, did the clinicians actually use it (which is what I think you were discussing in p.6, lines 41-42, but is unclear to me upon re-reading)? This latter point is also mentioned in the discussion but seems like it would be important to include as a result.
Thank you for your valuable feedback. This is a very good point. In the revised manuscript, we provide more details on how the visualization tool was presented and how it was used. In the table in the appendix, we summarize some qualitative data on whether the intervention tool was introduced to doctors and how doctors could access it, such as directly attached to an email or integrated into the EHR. Regarding whether clinicians use the visualization tool, we think this is a potentially very important factor affecting effectiveness. However, as mentioned in the original manuscript, there is limited evidence on this in the studies we included.

-In the discussion, there is mention about grouping into the 3 strategies (p. 8, lines 31-32), which seems to refer to a single sentence in the Results (p. 6, lines 31-38). If you feel these truly are important pragmatic groupings, perhaps the discussion should be expanded or refocused to include how you think these groupings might (or might not) be distinct from how or why a visualization might be effective and/or integrated into clinical care or operations.
Thanks again for your helpful comment. After discussion with clinicians, we grouped the results into these categories based on clinical behaviors and points in the delivery process. Our aim was to provide a practical framework to better understand how different types of visualization are used in clinical practice. However, we recognize that the number of studies we included was too small to support a robust analysis of these groupings. Therefore, we present the classifications as an attempt to summarize and categorize interventions rather than definitive groupings.

MINOR ISSUES

-As visualizations are the main focus of this paper, the current opening introductory paragraph is only loosely tied to the topic. If the thread to connect to the remainder of the paper is around visuals being part of audit and feedback strategies (as part of your discussion also suggests), perhaps that can somehow be integrated into the current second paragraph.
Thank you for your reply, which is a very helpful suggestion. We have revised the introduction to ensure a clearer narrative thread and enhance the coherence of the introduction.

-The Methods would be improved with a stricter adherence to the PRISMA guidelines. For example, the dates of searches should be included, whether the reviews were performed in duplicate, etc.
We appreciate your reminder and would like to clarify that we have addressed the concerns you mentioned. Specifically, the detailed search strategy (including search dates) has been provided in the Supplementary Materials. As described in the manuscript and shown in the selected figures, we removed duplicate records during the review process. In addition, we have included the PRISMA checklist in the Supplementary Materials to ensure transparency and adherence to the guidelines. We hope this clarifies that we have carefully followed the PRISMA guidelines. Thank you again for your thorough review and for helping us ensure that our manuscript meets high methodological standards.

-Was there any protocol registration and/or IRB approval for this study?
No institutional review board approval was sought since this was a secondary analysis of published work.

-Figure 1 should probably include separate exclusions at the title/abstract and the full text levels.
Thanks for your comment. We have updated it.
*** ***

*** ***

NEW REVIEWER COMMENTS (ON REVISED MANUSCRIPT):

Reviewer: 2
Dr. Lenore de la Perrelle, Flinders University, Flinders University
Comments to the Author:
Thank you for reviewing the manuscript. It appears that not all comments are responded to in comments.
The revised manuscript would benefit from a few minor terminology and wording changes to clarify further.
Thank you for taking the time to review our manuscript and provide valuable feedback. It seems that our previous response did not address all comments. When I downloaded the decision letter from the submission system, I

could only see some of the reviewers' comments in the letter for unknown reasons. It seems that there was an issue that caused your valuable feedback to not be included in the file I received. I apologize for any inconvenience this may have caused. We have considered all your insightful suggestions and comments and made necessary revisions to improve the quality of our manuscript. Thank you for your understanding and patience. We greatly appreciate your contribution to enhancing our work.

Abstract:L12 'in general' is still not clear. Consider rephrasing to 'overall effectiveness"
L14 would be useful to add 'this review addresses the gap in evidence for the overall effectiveness of visual display performance interventions'
Thanks for your comment, which is very helpful. We have revised.

Introduction
p 5 L46-50 rephrase the descriptions to avoid repeated use of like. Consider 'focus on type (scorecard or dashboard) or setting (primary health or aged care).
Specify the gap in literature: To address the gap in literature about the effects of ... display tools on ... we conducted...'
Thanks for your comment, which is very helpful. We have revised the introduction section to clearly articulate the research gap and rephrased the descriptions.

Methods
p6 L9-16 clarify dates and if a protocol was used. Short sentences may be better linked
Thanks for your comment. "We searched from these libraries respective inception dates and searches last updated on December 2022."

Discussion p9 L56 indicates studies published 2020-2021, not 3 years as noted in comments responding to reviewer. need to confirm
Thanks, it should be 2 years it is a typo.

p10 L9-22 a reflection on the value of a mixed methods review to capture additional components of an intervention may be useful and included in recommendations for further research. Many QI and behaviour change interventions would identify reasoning of clinicians in whether to engage in the intervention or why.
Thx for your comment. It is a really good point. Prior qualitative research and quality improvement (QI) studies have explored clinicians' attitudes engagement, which is critical to understanding the full impact of these interventions. Our study focused on evaluating effectiveness, and therefore, we did not delve into why engage in the intervention. We look forward to future studies that employ more mixed methods to better capture the complexity of clinician engagement. By combining quantitative data with qualitative insights, key factors that influence engagement and effectiveness can be identified.

p12 L42-53 this section is not clear and needs a review and a reference. is it better placed in the discussion earlier than in limitations? does this mean that the effectiveness measures alone do not indicate the mechanism of change and that exploration of this is needed?

We apologize for any confusion caused in the relevant section. We understand the concern about whether effectiveness measures alone indicate mechanisms of change. We intended to show that while effectiveness measures reflect the results of the visualizations, they do not necessarily reveal the underlying mechanisms driving those results. In our study, we aimed to transform various visualizations into uniform effect size measures for easier comparison. However, due to differences in outcome measures across studies and the limited number of articles available, we were unable to effectively group or compare these measures. Therefore, we did not have

sufficient data to explore or elucidate mechanisms of change. We appreciate your understanding and agree that exploring these mechanisms is important for a more comprehensive understanding, but given the data limitations, our study was limited to robustly assessing effectiveness.

Reviewer: 4
Dr. Joel Boggan, Durham Veterans Affairs Medical Center
Comments to the Author:
[NOTE FROM THE EDITOR: The reviewer has declined to submit a re-review at this time because the authors have not responded to any of their previous comments]

Thank you for taking the time to review our manuscript and provide valuable feedback. It seems that our previous response did not address all comments. When I downloaded the decision letter from the submission system, I could only see some of the reviewers' comments in the letter for unknown reasons. It seems that there was an issue that caused your valuable feedback to not be included in the file I received. I apologize for any inconvenience this may have caused. We have considered all your insightful suggestions and comments and made necessary revisions to improve the quality of our manuscript. Thank you for your understanding and patience. We greatly appreciate your contribution to enhancing our work.

Reviewer: 3
Dr. Søren Knudsen, Aalborg University, Psychiatry in Region North Jutland
Comments to the Author:
Thank you for sending the revised edition. I appreciate the improvements made; however, there are still some areas that require attention before publication. Primarily, the introduction needs further revision to clarify the review's objectives clearly.

Abstract:
Please rewrite the background section to succinctly explain the relevance of the team, identify gaps in existing knowledge, and outline how this review aims to fill these gaps.
Thanks for your comment, which is very helpful. We have revised.

Introduction:
The introduction requires a thorough rewrite to clarify the purpose of the review. The text between lines 3 and 10 is particularly vague. It is essential to explain why the chosen theme is relevant, what existing knowledge covers, what gaps remain, and how this paper will address these gaps. Moreover, the link between Quality Improvement (QI) and clinicians' behavior, as mentioned in line 3, needs to be clarified. The role of audit and feedback in this context is also unclear. Additionally, if the three objectives listed at the end of the introduction are to be retained, they must be directly addressed in the discussion section or else removed.
Thanks for your comment, which is very helpful. We have revised the introduction section to clearly articulate the research gap.

Specific textual revisions:

On Page 6, line 11, please adjust to past tense.
Thanks. Have changed.

On Page 7, lines 9-16, cite the relevant references in the “risk of bias” section.
Thanks. Have changed.

On Page 7, lines 54-59 ("it is noteworthy…"), the text feels overly discursive. Consider moving this part to the discussion section or revising it to present the results more neutrally.
Thanks. Have changed.

VERSION 3 - REVIEW

**Reviewer**	**2**
**Name**	**de la Perrelle, Lenore**
**Affiliation**	**Flinders University, of Education, Psychology and Social Work**
**Date**	**25-Aug-2024**
**COI**	**None to declare**

---

a couple of minor revisions are needed

p 8 L5 needs rewording for clarity

P1 L 54 needs to be included in the conclusion p11-12

**Reviewer**	**3**
**Name**	**Knudsen, Søren**
**Affiliation**	**Aalborg University, Danish Center for Health Services Research**
**Date**	**20-Sep-2024**
**COI**	**None**

The article has been improved through the revision process. The text is now more coherent and easier to read. Therefore, I do not have any changes to suggest for the main text.

However, the abstract is still somewhat disorganized and imprecise.

In the "Background" section, the influence of contextual factors is mentioned, but these are not discussed in the main text. The purpose should be clearly stated, which might be better conveyed with the sentence provided in the Introduction: "Therefore, we conducted a systematic literature review to appraise the evidence for the effectiveness of interventions involving all visualization tools of clinician performance display intended to improve process and outcome care"

The sentence, “Relatively more articles were published in the last two years on the effectiveness of dashboards,” is not particularly relevant. It is unclear what it is relative to, and there are other findings that are more pertinent and should be prioritized.

The punctuation could be improved, especially in the Results section.

The sentence, “This review focuses on evaluating the overall impact of a performance visualization intervention on clinician behavior change to improve healthcare quality,” does not belong under “strengths and limitations of this study.”

If the abstract is improved, I believe the article could be accepted for publication.

**Reviewer**	**4**
**Name**	**Boggan, Joel**
**Affiliation**	**Durham Veterans Affairs Medical Center**
**Date**	**29-Aug-2024**
**COI**	**n/a**

No further required comments at this time.

My main thought from the previous author responses is that the inclusion of the forest plot may not be as clear as the authors intend. I understand this figure was not included as part of a meta-analysis and I agree that a further quantitative analysis should not have been undertaken; however, the likely very significant heterogeneity in the purposes of the included studies, the clinical scenarios, and the often unclear use/implementation of their studied visual tools may make any visual representation of their results somewhat misleading for the casual reader compared to a narrative synthesis alone.

VERSION 3 - AUTHOR RESPONSE

Reviewer: 2
Dr. Lenore de la Perrelle, Flinders University, Flinders University
Comments to the Author:
a couple of minor revisions are needed
p 8 L5 needs rewording for clarity
P1 L 54 needs to be included in the conclusion p11-12

Thank you for this feedback. We have edited p8 L5 for clarity and believe this now reads better.

Regarding the conclusion in the abstract and the main text conclusion, we have also adjusted the wording to harmonize the messages.

Reviewer: 4

Dr. Joel Boggan, Durham Veterans Affairs Medical Center
Comments to the Author:
No further required comments at this time.

My main thought from the previous author responses is that the inclusion of the forest plot may not be as clear as the authors intend. I understand this figure was not included as part of a meta-analysis and I agree that a further quantitative analysis should not have been undertaken; however, the likely very significant heterogeneity in the purposes of the included studies, the clinical scenarios, and the often unclear use/implementation of their studied visual tools may make any visual representation of their results somewhat misleading for the casual reader compared to a narrative synthesis alone.

Thanks for your comment. After carefully considering about your suggestion, which we think is a good point, we have moved this forest plot to the supplementary material, added the results of the outcome variable in Table 1, and revised the related context.

Reviewer: 3
Dr. Søren Knudsen, Aalborg University, Psychiatry in Region North Jutland
Comments to the Author:
The article has been improved through the revision process. The text is now more coherent and easier to read. Therefore, I do not have any changes to suggest for the main text.

However, the abstract is still somewhat disorganized and imprecise.

In the "Background" section, the influence of contextual factors is mentioned, but these are not discussed in the main text. The purpose should be clearly stated, which might be better conveyed with the sentence provided in the Introduction: "Therefore, we conducted a systematic literature review to appraise the evidence for the effectiveness of interventions involving all visualization tools of clinician performance display intended to improve process and outcome care"

The sentence, "Relatively more articles were published in the last two years on the effectiveness of dashboards," is not particularly relevant. It is unclear what it is relative to, and there are other findings that are more pertinent and should be prioritized.

The punctuation could be improved, especially in the Results section.

The sentence, "This review focuses on evaluating the overall impact of a performance visualization intervention on clinician behavior change to improve healthcare quality," does not belong under "strengths and limitations of this study."

If the abstract is improved, I believe the article could be accepted for publication.

Thanks for your comment. Considering the editor's suggestions on the structure and your kind suggestions, we have revised the abstract.

*** ***

COI statements:

Reviewer: 2
Competing interests.: None to declare.

Reviewer: 4
Competing interests.: n/a.

Reviewer: 3
Competing interests.: None.
